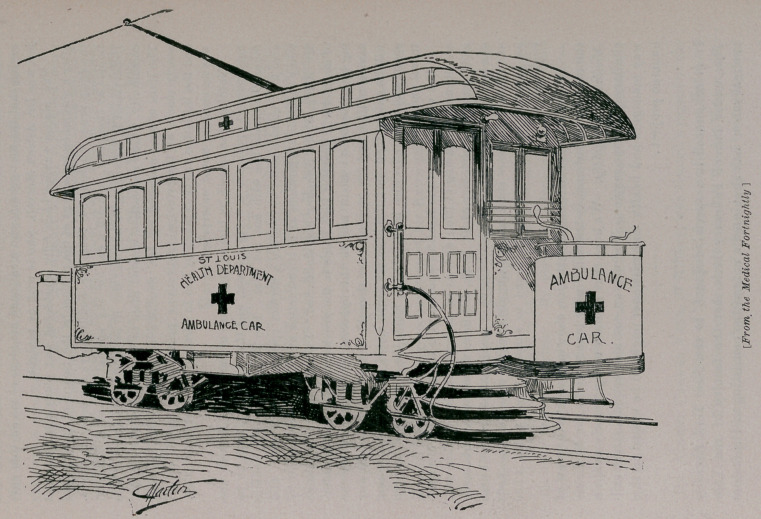# Topics of the Month

**Published:** 1895-03

**Authors:** 


### TOPICS OF THE MONTH.

A DESERVED rebuke has been administered to the Chicago Summer School of Medicine by the *Indiana Medical Journal.* The school referred to has, through its dean, Dr. William F. Waugh, issued a circular to physicians, stating that the college has placed at the disposal of the dean, a number of scholarships to enable students of limited means to be admitted to the classes at a material reduction from the regular fees. The circular invites physicians to furnish the names of "any such gentlemen," and affirms that the courtesy will be appreciated.

The *Indiana Medical Journal* says that it had hoped that this underhand bidding for students was about extinct among reputable colleges, and declares that institutions that have two prices are not in good standing and should be discountenanced.

The *Cincinnati Lancet-Clinic*, too, takes a hand in this criticism, and affirms that such bidding for students on the part of any medical school is reprehensible and should exclude an institution pursuing this course from membership in the American Medical College Association.

We quite agree with our contemporaries in their castigation of the course pursued by the Chicago school. It is a sign of healthy improvement when medical journals of standing and character speak out in unmistakable terms in condemnation of such reprehensible practices.

---

IN PREVIOUS issues of the JOURNAL we have alluded to the electric railway ambulance adopted by the city of St. Louis for the transportation of sick and wounded. The *Medical Mirror* for January, 1895, gives an account of the inauguration of this service, which was celebrated by a dinner given by Dr. George Homan, health commissioner, Thursday evening, December 27, 1894, to a number of the city officials, officers of various railroads and a select party of physicians and friends. The *Mirror* says:

> The occasion was one long to be remembered. The early part of the evening was taken up by a trial trip from the City Hall to the City Hospital and remoter points. The guests were filled with admiration for the beauty and general efficiency of the interior finish and the ease with which the car was carried.
>
> In the next issue of the *Medical Mirror* we shall present illustrations of the interior and exterior of this car, together with a description of the same.

Some one has well said: "He who maketh two blades of grass to grow where one has grown before, is a benefactor to the state," but it may truly be said that he who adds to the sum of pleasure and relief from pain of the unfortunates of the world, is indeed more than a benefactor to the state. He is a friend of humanity.

After the trial trip the guests assembled in the superb dining room of the Union Station, and such a dinner one rarely has the opportunity of enjoying. At the proper time Dr. I. N. Love, who had previously been invited to serve as toastmaster, rapped the company to order. Numerous congratulatory and entertaining speeches were made, notably by the Hon. Charles Nagel, president of the city council; Capt. John Scullin, Dr. Otto E. Forster, member of the board of health; Dr. James Moores Ball, editor of the *Tri-State Medical Journal*, and many others.

Dr. George Homan, health commissioner, the host of the evening, having been called upon, responded as follows:

*Mr. Toastmaster and Gentlemen*—Every step forward, be it great or small, must be counted in the life of a city in truly reckoning from time to time the progress it is making toward better things. And better things are those which show in themselves a real gain over the past, whether in mind, heart or soul, in science, intellect or work, in enlightened skill or humane achievement.

The occasion which brings this company together tonight is one that may, not unfairly, be held to partake of this character, small though its particular subject may be and far distinct from the personal sphere and daily life of many of those now present here.

The step forward which we mark this evening is in the line of more considerate treatment and more humane handling of sick and wounded unfortunates; and, if the worth of the civilization of today shall be measured in this wise and by such a standard, who, knowing the truth, will dare deny that our shortcomings are many and opportunities for improvement correspondingly great? Nor does it avail to extenuate our delinquencies in this regard by pleading that others have done no better.

St. Louis, as the metropolis of the Mississippi Valley, should be as a city set on a hill in all intelligent effort, conspicuous for excellence in every domain, an exemplar and a shining light to all the world.

The origin in me of the idea whose embodiment you have seen tonight, was the use of street cars and railways for United States mail purposes, this application having been realized through the intelligence and zeal of our then postmaster, Mr. Harlow; and, being quite familiar with the methods and equipment of the local Health Department, the thought of a car ambulance arose in my mind, and the same thought

[From the Medical Fortnightly]

must have been suggested to other minds also that had been bent to the question, as the time was ripe for changes, and I take pleasure in saying that the first person who mentioned his ideas to me on this subject was Dr. Albert Merrell, who is with us tonight.

The matter having been fully considered and plans outlined, and after a cordial endorsement of the project by the Board of Health and the daily press, on June 22, 1894, Mr. Scullin gave the order to the St. Louis Car Company, to build a car for the special use of the Health Department in conveying to hospital the ill or injured members of this community, whose necessities compel them to become a public charge. The lines of road operated by Mr. Scullin's company touch, or very nearly reach, all the principal institutions provided for public relief, and the initiative was thus taken by him in what, to my best knowledge, was an entirely novel enterprise.

Nothing was spared by him in time, care or means to furnish the most perfect appliance in every respect, and if there is any fault to be found the blame lies with me, not him. The builders of the vehicle also took infinite pains to produce a perfect specimen of its kind, and many valuable suggestions as to construction and detail were made by Mr. Kling, of the company just mentioned. I believe that this change of method will prove of such value in the prevention of needless suffering by patients in transit, and in the opportunities presented for the saving of limb and life, that no more thought of a return to the old way will be entertained than the business world would think of abandoning steam and electricity for horseflesh to meet the demands of modern travel and traffic.

The whole aim of the Health Department is the prevention of physical ills and the saving of life ; and, as an instrumentality to this end, and for the greater glory of St. Louis, I believe the electric ambulance car will have a place in the conduct here of municipal affairs until time and onward progress shall reveal to inquiring minds means and methods as superior to it as it will be found to surpass those which it has replaced.

We present herewith an illustration showing an exterior view of the electric ambulance in transit, kindly loaned us by the *St. Louis Medical Fortnightly*. We hope that Buffalo will promptly adopt a similar ambulance service. The mayor, common council, street railway managers and health commissioner ought to be united in this humane project.

---

It is announced in a special dispatch to the *New York Evening Post*, under date of February 12th, that the faculty of the Yale medical school has appointed a committee consisting of Professors

Smith, Carmalt and Ferris to prepare a plan for an extension of the three years' course of the school to four years. It is expected that the scheme will be perfected in season for announcement at the next commencement.

From advance sheets of the regents' annual report, we learn that of the 140 medical schools in the United States, there are now fourteen that absolutely require a four years' course of medical lectures; nearly 100 schools announce that they graduate on three terms of lectures and about twenty-five on two terms, the length of the term varying from five to nine months.

## Figures and Tables

**Figure f1:**